# Co-Occurrence of Defoliating and Non-Defoliating Pathotypes of *Verticillium Dahliae* in Field-Grown Cotton Plants in New South Wales, Australia

**DOI:** 10.3390/plants9060750

**Published:** 2020-06-15

**Authors:** Duy P. Le, Aphrika Gregson, Thao T. Tran, Rodney Jackson

**Affiliations:** 1 New South Wales Department of Primary Industries, Narrabri, NSW 2390, Australia; aphrika.gregson@dpi.nsw.gov.au (A.G.); rod.jackson@dpi.nsw.gov.au (R.J.); 2 Biology Department, School of Education, Can Tho University, Ninh Kieu 900000, Vietnam; tranthao2009@gmail.com

**Keywords:** *Gossypium hirsutum*, Verticillium wilt, pathogenecity, pathotyping, duplex PCR

## Abstract

Verticillium wilt (VW) is a major constraint to cotton production in Australia and worldwide. The disease is caused by a soilborne fungus, *Verticillium dahliae*, a highly virulent pathogen on cotton. Commonly, *V. dahliae* is designated into two pathotypes: defoliating (D) and non-defoliating (ND), based on induced symptoms. In the previous two survey seasons between 2017 and 2019, stems with suspected VW were sampled for the confirmation of presence and distribution of D and ND pathotypes across New South Wales (NSW), Australia. A total of 151 and 84 VW-suspected stems sampled from the 2017/18 and 2018/19 seasons, respectively, were subjected to pathogen isolation. Of these, 94 and 57 stems were positive for *V. dahliae*; and 18 and 20 stems sampled respectively from the two seasons yielded the D pathotype isolates. Two stems from the 2017/18 season and one stem from 2018/19 season yielded both D and ND pathotype isolates. We also successfully demonstrated the co-infection of both pathotypes in pot trials, which was driven predominantly by either of the pathotypes, and appeared independent on vegetative growth, fecundity and spore germination traits. Our study is the first report of the natural co-occurrence of both D and ND pathotypes in same field-grown cotton plants in NSW, to which a challenge to the disease management will be discussed.

## 1. Introduction

Cotton (*Gossypium* spp.) is an economically important fibre crop contributing to approximately 40% of the world’s natural fibre [1]. Cultivated cotton can be found in a range of ecological niches from arid to semi-arid areas of the tropical and subtropical zones and is mainly derived from Upland cotton (*G. hirsutum* L.) and Pima cotton (*G. barbadense* L.) due to superior fibre quality and quantity traits [2]. In Australia, Upland cotton is a major agricultural crop and mainly grown in regional areas of New South Wales (NSW) and Queensland and produced predominantly under irrigated conditions, but smaller areas of dryland production do occur in some seasons [3]. In the 2017/18 cotton growing season, the industry employed up to 10,000 people across 152 communities and contributed significantly to the economic growth and wealth of these regions. The majority of Australian cotton is exported, which generates an average value of around AUD 1.9 billion annually [3].

In Australia, cotton is prone to infection with an array of pathogens, including black root rot pathogen *Thielaviopsis basicola* (Berk. and Br.) Ferraris [4], leaf spot pathogen *Alternaria alternata* (Fr.) Keissl. [5], Fusarium wilt pathogen *Fusarium oxysporum* f. sp. *vasinfectum* Snyder and Hansen, [6,7], and Verticillium wilt (VW) pathogen *Verticillium dahliae* Kleb. [8]. Of these, VW of cotton is of a major constraint to the sustainability of the cotton industry due to the lack of complete resistant resources and highly effective management strategies. The disease is associated with vascular discolouration, leaf chlorosis, necrosis, defoliation and plant death in some severe cases [9]. In NSW, VW incidence in cotton was as little as 3% up to 16% on average between 1984 and 2012 [8]. During 2016–2019, the highest average incidence of VW in NSW and Queensland was 30% and 4%, respectively [10]. According to Allen [11], yield loss caused by the disease in Australia can be up to 25% in years where climatic conditions favoured disease development. On a rare occasion, yield loss was estimated up to 50% in a severely infested field in NSW (unpublished data).

*Verticillium dahliae* is a soilborne phytopathogen and commonly associated with vascular wilt diseases of up to 400 host plant species; many of these are of economic importance in agriculture, horticulture and forestry [12]. *V. dahliae* was reported across many cotton growing regions such as Australia, China, Spain and the USA [8,13,14,15]. Virulence of *V. dahliae* was commonly found associated with its pathotypes, that being defoliating (D) and non-defoliating (ND), based on symptoms induced on host plants [16,17]. Unless otherwise stated, the D and ND pathotypes should only be referred to as pathotypes of *V. dahliae* in this study. The D pathotype was deemed to be highly aggressive, inciting defoliation, and was lethal to cotton; alternatively, the ND pathotype was considered less aggressive and did not attribute to defoliation [13]. However, *V. dahliae* isolates recovered from Australian cotton and designated as D and ND pathotypes were found to be equally lethal to its host (unpublished data). In the past two survey seasons, the ND pathotype was detected across NSW, while the D pathotype was more prevalent in the northern valleys of NSW [10]. Additionally, our initial data also indicated the presence of both pathotypes in some fields in NSW (unpublished data). Co-occurrence of both pathotypes in a cotton field were previously reported in Spain [16]. Similarly, Jiménez-Díaz et al. [18] found that co-occurrence of both pathotypes was relatively common in olive orchards in Spain. Interestingly, Mercado-Blanco et al. [19] for the first time demonstrated that co-infection of D and ND pathotypes also occurred naturally in olive trees. This reflects the complexity of the patho-system in *V. dahliae*. However, many used single isolates of *V. dahliae* for biological and pathogenicity assessments [20,21].

Interactions between *V. dahliae* with either other *Verticillium* spp. or different isolates were assessed in artificially co-inoculated assays. The observed effects varied from none to cross-protection, depending on the inoculation methods, orders and time intervals between inoculations [22,23]. For example, VW expressions on lettuce were lower in co-inoculated plants with isolates of *V. tricorpus* and *V. dahliae* compared with those inoculated with *V. dahliae* alone. Additionally, the co-inoculation relatively promoted the growth of lettuce [23]. Qin et al. [23] also reported that a soil drench with *V. tricorpus* in advance of the inoculation with *V. dahliae* appeared to provide better protection compared with simultaneous inoculation using a root-dip method. On the other hand, simultaneous inoculation of the avirulent isolate P6 and the highly virulent isolate VM of *V. dahliae* did not result in any difference in disease expression on sunflower; however, sequential inoculation of the isolate VM two days following the challenge with the isolate P6 resulted in a significantly lower disease severity in sunflower [24]. Shittu et al. [25] found that disease scores in tomato were significantly lower in plants either preceding inoculation with the non-host isolate Dvd-E6 followed by the virulent isolate Vd1 of *V. dahliae* or in simultaneous inoculation. Wheeler and Johnson [22] reported that co-inoculation with two or three different host-selective *V. dahliae* isolates did not alter potato yields, or mustard and barley biomass, but disease severity in potato increased in co-inoculated plants. On cotton, cross-protection was observed in plants grown both under artificially co-inoculated and naturally infested soils with the virulent D pathotype isolate T-1 and mildly virulent ND pathotype isolate SS-4 [26]. However, at the time, there was no evidence that supported the co-existence of both pathotypes in cotton plants [26]. To date, only Mercado-Blanco et al. [19] showed the natural co-infection and co-existence of the D and ND pathotypes in olive. Therefore, our study sought to document for the first time the natural co-occurrence of D and ND pathotypes in field-grown cotton sampled in NSW, Australia, which were also demonstrated again in our co-inoculated pot trials.

## 2. Results

### 2.1. Isolation and Pathotype

A total of 151 and 84 VW-suspected stems selected from samples collected during the 2017/18 and 2018/19 seasons, respectively, were subjected to recovery of *V. dahliae* (Table 1). Of these, 94 and 57 stems from the two seasons, respectively, yielded putative *V. dahliae* cultures on an isolating medium (potato dextrose agar amended with 100 ppm of streptomycin, sPDA). Subsequently, a total of 195 and 120 putative *V. dahliae* isolates from the 2017/18 and 2018/19 seasons, respectively, were subcultured and purified for pathotyping using duplex PCR developed by Mercado-Blanco et al. [19]. Of these putative *V. dahliae* isolates, 34 and 41 isolates accounting for approximately 17% and 34% accordingly were designated to the D pathotype (Table 1), thus confirming the identification of the pathogen.

Of the *V. dahliae*-positive stems, 18 and 76 stems were determined to yield the D and ND pathotypes, respectively, in the 2017/18 season, and two stems yielded both pathotypes. In the 2018/19 season, 20 out of the 57 *V. dahliae*-positive stems yielded the D pathotype, and one yielded both the D and ND pathotypes. Therefore, this confirms the first report of the natural co-occurrence of both the D and ND pathotypes of *V. dahliae* in cotton in NSW, Australia.

### 2.2. Pathogenicity

#### 2.2.1. Trial 1

Control plants remained healthy (disease incidence and severity = 0) during the time course of the experiment; hence, unless otherwise stated, the control data were excluded in our ANOVA analyses and graphing.

**Figure 1:**
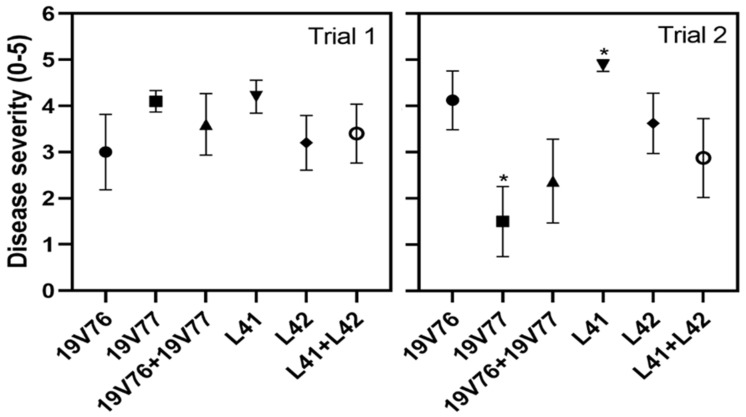
Mean disease severity (0: no symptoms–5: dead plants) recorded at four weeks after root dip inoculation of 2-week-old seedlings either singly or in combination with 10^4^ spores/mL suspensions of the tested *V. dahliae* isolates. Bars represent standard errors of means (*n* = 10 and 8 in trial 1 and trial 2, respectively). Asterisks indicate significant differences among treatments (*p* = 0.05).

Plants inoculated with the tested *V. dahliae* isolates showed the first VW symptoms, including chlorosis and wilting on cotyledons seven days after inoculation. Disease symptoms progressed upwards, and defoliation was observed on plants inoculated with isolates 19V76 and L42, and those with co-inoculation (Table 2). We recorded various degrees of disease severity from 0 = no symptoms to 5 = dead plants four weeks after inoculation. The mean disease severity varied from 3 to 4.2 and was not significantly different among the *V. dahliae*-inoculated treatments, irrespective of either single- or co-inoculation (Figure 1).

Of the diseased plants, the *V. dahliae* recovery frequencies of the inoculated isolates were from 71 to 100% (Table 2). We failed to recover any *V. dahliae* from asymptomatic inoculated plants and the control plants. A single pathotype, either D or ND, was recovered from the single inoculated plants. Both D and ND pathotypes were recovered only from the co-inoculated plants. Two plants from each of the co-inoculated treatments were co-infected with both pathotypes. The co-infections were driven by either of the pathotypes, indicated by the predominant recovery of one to the other (Table 2).

#### 2.2.2. Trial 2

No VW disease symptoms were observed on the control plants. The first VW symptoms were noted on cotyledons of the *V. dahliae*-inoculated plants at nine days after inoculation. Defoliation was observed on plants inoculated with isolates 19V76 and L42 and their co-inoculation at four weeks after inoculation (Table 2). Disease incidence was lowest (38%) in the 19V77 treatment and highest (100%) in the L41 treatment. Means of disease severity were significantly different (*p* = 0.05) between the 19V77 and L41 treatments, but these were not different to the others (Figure 1).

Of the diseased plants, the *V. dahliae* recovery frequencies of the inoculated isolates ranged from 61 to 100% (Table 2). As in trial 1, we did not recover any *V. dahliae* from asymptomatic inoculated plants and the control plants. Diseased plants from treatments singly inoculated with either D or ND yielded a single pathotype upon isolation and pathotyping using duplex PCR. Both D and ND pathotypes were recovered only from the co-inoculated plants. A single plant from the co-inoculated L41 + L42 treatment yielded both D and ND pathotypes. The co-infection was driven by the ND pathotype; six out of seven recovered isolates were designated to the ND pathotype (Table S1).

### 2.3. Growth Competition

**Figure 2:**
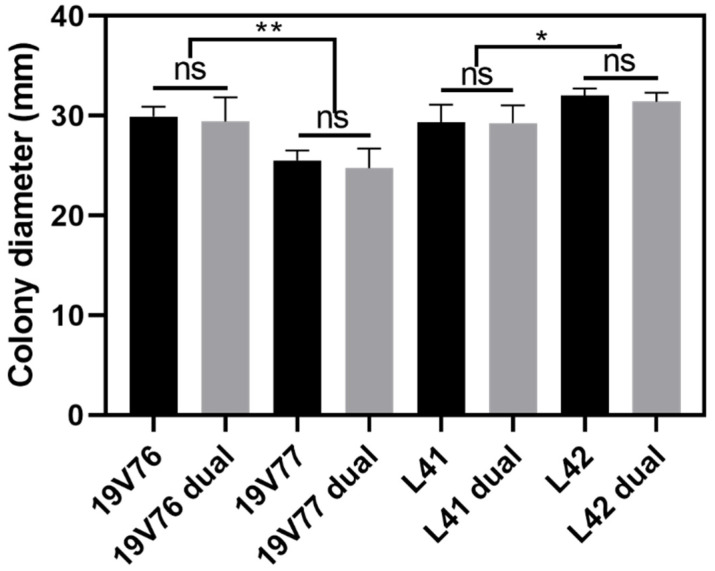
Colony diameter (mm) of the tested *V. dahliae* isolates recorded after seven days growing in darkness at 25 °C. Growth competition of 19V76 vice versa 19V77, and L41 vice versa L42 were assessed in dual culture assays (labelled “dual”). Data from the two assays were pooled due to the insignificant difference found between the two repeated assays. Bars represent standard errors of means (*n* = 12). Asterisks indicate significant differences among treatments (*p* = 0.05 for * and *p* = 0.01 for **).

At 25 °C, the D isolates 19V76 and L42 grew respectively faster than the ND isolates 19V77 and L41 after seven days incubation in darkness, regardless of being grown in single or dual cultures (Figure 2). Colonies of 19V76 single and 19V76 dual (against 19V77) reached approximately 30 mm in diameter and were significantly higher than those of 19V77 single and 19V77 dual (against 19V76), which were around 25 mm in diameter. Similarly, growth of L42 single and L42 dual (against L41) were about 10% faster than those of L41 single and L41 dual (against L42).

There were no negative effects on growth of the tested *V. dahliae* isolates recorded in the dual culture assays (Figure 2). Growth of 19V76 in single culture (29.9 mm diameter) was comparable to that of the dual culture against 19V77 (29.4 mm diameter). A similar pattern was recorded on the growth of 19V77, L41 and L42 in single and dual cultures. This indicates growth competition between the D and ND isolates was not observed in our assays.

### 2.4. Fecundity and Germination

**Figure 3:**
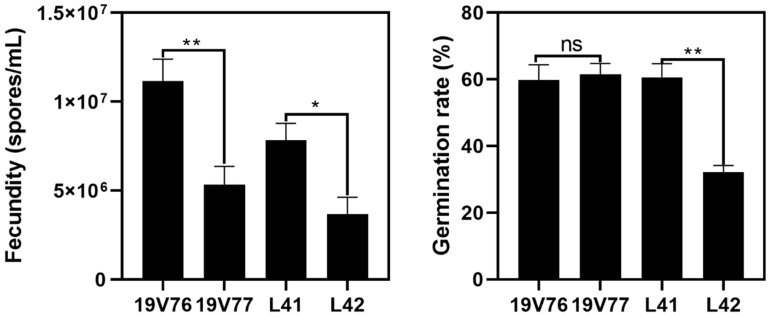
Fecundity (spores/mL) and germination rate (%) for the four tested *V. dahliae* isolates on potato dextrose agar amended with 100 ppm of streptomycin (sPDA) at 25 °C in darkness. Data from the two assays were pooled due to the insignificant difference found between the two repeated assays. Bars represent standard errors of means (*n* = 6 and 18 for fecundity and germination assessments, respectively). Asterisks indicate significant differences among treatments (*p* = 0.05 for * and *p* = 0.01 for **).

The in vitro fecundity (spores/mL) and germination rate (%) of the four tested *V. dahliae* isolates were isolate-dependent, but independent from pathotypes (Figure 3). Fecundity of the D isolate 19V76 was around 1.1 × 10^7^ and significantly higher than that of the ND isolate 19V77 (5.3 × 10^6^). On the other hand, the ND isolate L41 produced a significantly higher number of spores compared with the D isolate L42 under the same conditions.

There was no significant difference in the spore germination rates of the 19V76 and 19V77 isolates on sPDA, which were recorded around 60%. Conversely, the germination rate of L42 was recorded at 32.2%, which was about half of L41 (60.6%) (Figure 3).

## 3. Discussion

Verticillium wilt of cotton was first reported in NSW in 1959 [27]. Over 35 years of continuous disease survey since 1984 [8], we for the first time documented the natural co-occurrence of both D and ND pathotypes of *V. dahliae* in cotton in NSW, Australia, sampled from two survey seasons between 2017 and 2019. Additionally, we were able to demonstrate the co-infection in two independently repeated pot trials using a root dip method. Therefore, we propose that co-infections of multiple *V. dahliae* isolates in a cotton plant are probably not a rare event under natural field conditions. Previously, Mercado-Blanco et al. [19] reported the co-infection of D and ND pathotypes occurred naturally in olive trees. Schnathorst and Mathre [26] artificially co-inoculated cotton with the virulent D isolate T-1 and mildly virulent ND isolate SS-4; however, there was no evidence of co-infection recorded in their study.

Our pot trials indicated that the D and ND isolates recovered from the same stem were relatively comparable in virulence on cotton, and the VW disease expressions were not significantly different between the single- and co-inoculated plants. Irrespective of treatments, initial VW symptoms appeared first on cotyledons between 7 and 9 days after inoculation and dead plants were observed across the treatments four weeks after inoculation. Similarly, Wheeler and Johnson [22] reported VW disease in potato plants co-inoculated with three isolates from potato, mint and tomato was highly comparable to plants who received a single inoculation with the potato isolate. Interestingly, Schnathorst and Mathre [26] found cross-protection on cotton when co-inoculated with virulent D and mildly virulent ND isolates. Cross-protection was more often associated with co-inoculations of avirulent and virulent isolates. Shittu et al. [25] reported the VW disease scores were reduced by half on tomato plants co-inoculated with the highly virulent Vd1 and endophyte Dvd-E6 isolates compared with those recorded from single-inoculated tomato with Vd1. Sunflower challenged prior with the avirulent isolate P6 was protected from sequential inoculation with a virulent isolate of VM, *V. dahliae*; however, simultaneous inoculation of the two isolates did not result in different disease expressions [24].

We proposed that the D and ND isolates were able to colonise cotton stems equally due to the even number of isolates recovered from the same field-sampled stems. However, in our pot trials, the recovery frequency of D and ND isolates from the confirmed co-infected stems were predominated by one to the other. Mercado-Blanco [19] directly detected both D and ND isolates from co-inoculated olive roots at 0, 7 and 21 days after inoculation; however, DNA directly obtained from stems of the same plants were only positive with the D marker. Similarly, Shittu et al. [25] suggested that tomato plants colonised by the endophyte isolate Dvd-E6 ameliorated the effectiveness of colonisation of the pathogenic isolate Vd1. We postulated that vegetative growth competition may play a minor role in co-infection and co-colonisation of the tested *V. dahliae* isolates in co-inoculated plants since there were no negative effects of one to the other detected in the dual culture assays. Fecundity and the spore germination rate of the tested isolates could partly play a role in driving this predominant colonisation. For example, the isolate 19V76 produced significantly more spores than 19V77; subsequently, in the two pot trials, the isolate 19V76 was recovered more than the isolate 19V77 in co-inoculated plants. However, we did not see a similar pattern in plants co-inoculated with the L41 and L42 isolates, though L41 produced a higher number of spores which had double the germination rate compared with L42. It is not possible to offer any conclusive recommendations from the current data set since host plant responses also play an important role in a successful colonisation of the pathogen. It will be worth using green fluorescent protein (GFP)-tagged isolates to better understand the interactions between *V. dahliae* isolates as well as with the host in planta. GFP-tagged *V. dahliae* was studied to understand its capacity to colonise cotton cultivars with different degrees of susceptibility [28].

The occurrence of both D and ND pathotypes within a single cotton plant under field conditions has also raised concerns regarding the development of disease management strategies. First, commercial Australian cotton germplasms exhibited varietal responses to D and ND pathotypes differently, and to date, there has not been a highly resistant cultivar against both pathotypes, especially to the D pathotype (C. R. Trapero per. comm.). Therefore, there will be limited cultivars available for *V. dahliae*-infested fields where the co-occurrence of both D and ND pathotypes in cotton plants were detected. However, before this raises an alarm, a thorough assessment of the damage that co-infection may cause should be pursued. The co-infection incidence under natural field conditions was detected at a low level in the previous two seasons. We continue to carry out disease surveillance, which has been ongoing for over 30 years in NSW, to enable the monitoring of the occurrence and distribution of the co-infection of both D and ND pathotypes on cotton. Second, genetic combination was also questioned. Wheeler and Johnson [22] recently reported the putative anastomosis in planta when mustard plants were co-inoculated with three isolates: potato 653, mint 111 and tomato 461. Therefore, understanding the diversity of the isolate collection recovered from co-infected cotton in this study will warrant future research.

## 4. Materials and Methods

### 4.1. Sampling and Isolating

During the 2017/18 and 2018/19 survey seasons, cotton stems from VW-suspected plants were sampled for further confirmation through isolation and identification of the actual causal agent. Where possible, at least three VW-suspected stem cuts, approximately 10–15 cm long, were sampled from each of the surveyed fields. These stem cuts were double-bagged inside a paper bag and another outside zip-lock plastic bag. The stem cuts were stored in an esky (a portable cooler) away from direct sunlight during the survey trips and immediately transferred to a 4 °C fridge/cold room after each trip until further processed.

Isolation of the putative pathogen was initiated by excising each of the stem cuts into smaller sections, 1–2 cm long, and peeling off the outer bark. Under aseptic conditions, each of the peeled sections was sprayed and left for 30 s with 70% ethanol for surface decontamination. The sprayed section was then plotted dry with paper towel and split in half using a sterile scalpel. Inner vascular discoloured tissues (wood chips) were thinly sliced with the scalpel and embedded into sPDA. sPDA was made of potato dextrose agar (PDA Difco) amended with 100 ppm streptomycin sulfate (Sigma Aldrich) and contained in Petri plates. The plates with embedded vascular tissues were sealed with parafilm and incubated at 25 °C in the dark for 3–5 days. Putative fungal colonies emerging from vascular tissues were individually sub-cultured onto new sPDA plates and single spore cultures were established. Pure cultures were then transferred onto half strength sPDA and incubated at 25 °C in darkness for at least a week before small plugs (0.5 cm^2^) were excised from the colony margins, submerged in sterile water and stored at room temperature for subsequent experimentation.

### 4.2. Pathotyping by Duplex PCR

#### 4.2.1. DNA Extraction

Genomic DNA was obtained using a Wizard^®^ Genomic DNA Purification Kit (Promega, Sydney Australia) following the manufacture’s protocol. However, slight modifications were deployed to suit our laboratory conditions. A small amount of mycelia (10–100 mg) was scraped off culture plates and transferred into a 1.5 mL tight-lock Eppendorf tube. Then, 50 µL Nuclei Lysis Solution was added along with two steel beads (3.3 mm dia.) to each tube and shaken to macerate the mycelia on a tissue lyser (Retsch^®^ MM300) for 1 min at a frequency of 28 times per second. Another 450 µL Nuclei Lysis Solution was then added to each of the tubes, vortexed to homogenicity and followed by incubation in a water bath at 65 °C for 30 min. After incubation, 3 µL RNase A Solution was added to each of the tubes and followed by another incubation at 37 °C for 15 min. After cooling down at room temperature for 5 min, 200 µL Protein Precipitation Solution was added and vortexed vigorously to homogenicity and followed by a centrifugation at 13,000 rpm for 5 min. The supernatants were carefully transferred to new 1.5 mL Eppendorf tubes containing 600 µL room temperature isopropanol. The tubes were gently inverted and centrifuged at 13,000 rpm for 1 min. The supernatants were carefully decanted and the visible DNA pellets were washed twice with 70% room temperature ethanol. The DNA pellets were then air-dried under a fume hood for 30–45 min, rehydrated with 50–200 µL DNA Rehydration Solution depending on the size of the pellets and followed with an incubation at 65 °C for 1 h. The DNA solutions were then stored at −20 °C until use.

#### 4.2.2. Duplex PCR Amplification

A duplex PCR assay developed by Mercado-Blanco et al. [19] was deployed to simultaneously characterise the two defoliating (D) and non-defoliating (ND) pathotypes. All PCR amplifications were carried out using GoTaq^®^ G2 Green Master Mix (Promega). Each PCR mix contained: 10 µL of Green Master Mix, 8 µL of DNase free water, 1 µL of 10 mM primer mix and 1 µL of DNA template. DNase-free water was included as a negative (no-template) control. The primer mix included 3 portions of DB19 (5′-CGGTGACATAATACTGAGAG-3′), 2 portions of DB22 (5′-GACGATGCGGATTGAACGAA3′) and 1 portion of espdef01 (5′-TGAGACTCGGCTGCCACAC-3′). PCR cycling conditions were slightly modified from Mercado-Blanco et al. [19] as follows: initial denaturation for 5 min at 94 °C followed by 35 cycles of 30 s at 94 °C, 30 s at 52 °C and 90 s at 72 °C, with a final elongation step of 7 min at 72 °C. The PCR products were run at 100 V in a GelRed (GeneTargetSolutions) pre-stained 1.5% agarose gel for 45 min and visualised under UV light using a UVIDOC HD6 (UVITEC Cambridge). D pathotype isolates were predicted to contain two visible PCR amplicons at sizes of 539 and 334 bp, whereas ND pathotype isolates contained a single amplicon at a size of 523 bp [19].

### 4.3. Pathogenicity

Root-dip assays were conducted twice in glasshouse conditions to assess the virulence of D and ND isolates solely and in combination. Two isolates from each of the pathotypes were selected for the pathogenicity assays (Table 3). Both D and ND isolates recovered from the corresponding year were isolated from a single stem.

Cotton black seeds (cv. Sicot 75RRF, a VW susceptible cultivar) were sown individually in a 100-cell plastic seedling tray containing Searles^®^ potting mix and grown in a glasshouse at 10–25 °C for 15 days prior to inoculation. Upon inoculation, seedlings were gently removed from the seedling trays and washed free of the potting mix. The washed seedlings were root-dipped in conidial suspensions of the four tested isolates at the concentration of 10^4^ conidia/mL for five minutes and then transferred into 140 mL pots containing Searles^®^ potting mix. There were two additional co-inoculum mixtures of L41 and L42, and 19V76 and 19V77. Conidial suspensions were mixed and adjusted to 10^4^ conidia each/mL. There were two seedlings per pot and five and four replicate pots for each of treatment, respectively, in trial 1 and 2. Control seedlings were treated in the same manner; however, the spore suspension was replaced with sterile water. Inoculated seedlings were maintained in the same glasshouse conditions and monitored for disease occurrence and severity. Disease ratings were as follows: 0 = no symptoms, 1 = chlorosis and wilting of cotyledons, 2 = chlorosis and wilting of first true leaf, 3 = symptoms on lower 50% of the foliage, 4 = symptoms on 51–100% of the foliage and 5 = dead plant.

Upon termination of the pathogenicity assays, all plants were subjected to re-isolation of the inoculated pathogens. The isolation of the inoculated *V. dahliae* from the collar sections was as described previously. All recovered isolates were also subjected to the duplex PCR again for determination of the pathotypes.

### 4.4. Growth Competition Assays

Relative growth of the four tested isolates was assessed singly at 25 °C. Briefly, actively growing *V. dahliae* cultures on sPDA were excised into 0.5 cm^2^ plugs and transferred into the centre of new sPDA plates. Additionally, a dual cultures technique was undertaken to assess the growth competition of the *V. dahliae* isolates under the influence of one to another. As previously, active cultures of L41 and L42, and 19V76 and 19V77 were excised, transferred in pairs together and placed 5 cm apart onto new sPDA plates. All newly transferred plates were sealed with parafilm and incubated in darkness at 25 °C for seven days before the colony dia. was recorded in perpendicular directions. There were three replicate plates per each isolate/pair and the whole assay was repeated once.

### 4.5. Fecundity and Germination Assessments

Ten-day-old cultures growing at 25 °C in darkness were subjected to spore collection for fecundity and germination assessments. There were three plates per isolate for the fecundity examination. Each plate was flooded with 10 mL of sterile water and gently interrupted with a disposable L-shaped spreader. The spore concentration per mL of the collected suspension was determined using a haemocytometer. The spore suspension was then adjusted to 10^4^ per mL for the germination assessment. Three 10 µL aliquots from each of the spore suspensions were individually spread onto clean sPDA plates (*n* = 9 per isolate). The plates were then incubated at 25 °C in darkness for two days, after which the colonies of *V. dahliae* were recorded. The experiment was repeated once.

Data collected from the pathogenicity, growth rate, fecundity and germination assays were subjected to an analysis of variance (ANOVA) and the separations of means were determined by Tukey’s least significant difference (LSD) test (*p* ≤ 0.05). The ANOVA and graphing were performed with Graphpad Prism 8.2.0.

## Figures and Tables

**Table 1:** Number of *V. dahliae* isolates including pathotype designations recovered from Verticillium wilt (VW) disease-suspected stems sampled during the two survey seasons between 2017 and 2019.

Numbers of	2017/18 Season	2018/19 Season
VW-suspected stems ^1^	151	84
*V. dahliae*-positive stems ^2^	94	57
ND-positive stems ^3^	76	37
D-positive stems	18	20
Both-pathotypes co-occurred stems	2	1
Putative *V. dahliae* isolates	195	120
D isolates ^4^	34	41
ND isolates	161	79

^1^ VW-suspected stems sampled during the late season disease surveys in the previous two seasons were subjected to isolation for confirmation of the associated pathogen. A minimum of three stems exhibiting typical VW peppery vascular discoloration were selected per field for pathogen isolation. ^2^ *V. dahliae*-positive stems were only confirmed once the corresponding pathogen was recovered. ^3^ D- and ND-positive stems were determined based on pathotyping results of the correspondingly recovered *V. dahliae* isolates. ^4^ D and ND isolates were designated using duplex PCR [19].

**Table 2:** Number of diseased, D and ND plants recorded in the two pot trials inoculated with the tested *V. dahliae* isolates, and the number of D and ND isolates recovered upon termination (four weeks after inoculation) of the pathogenicity assays.

Numbers of	Control	19V76	19V77	19V76 + V77	L41	L42	L41 + L42
**Trial 1 ^1^**							
Diseased plants ^2^	0	6 (60)	10 (100)	8 (80)	10 (100)	9 (90)	8 (80)
D plants ^3^	0	5	0	4	0	2	4
ND plants	0	1	10	4	10	7	4
D and ND positive plants ^4^	0	0	0	2	0	0	2
*V. dahliae* isolates ^5^	0	42 (100)	57 (71)	51 (91)	68 (97)	54 (86)	50 (89)
D isolates ^6^	0	42	0	31	0	54	18
ND isolates	0	0	57	20	68	0	32
**Trial 2**							
Diseased plants	0	7 (88)	3 (38)	4 (50)	8 (100)	7 (88)	5 (63)
D plants	0	7	0	1	0	5	4
ND plants	0	0	3	3	8	2	1
D and ND positive plants	0	0	0	0	0	0	1
*V. dahliae* isolates	0	49 (100)	21 (100)	17 (61)	56 (100)	45 (92)	25 (71)
D isolates	0	49	0	11	0	45	21
ND isolates	0	0	21	6	56	0	4

^1^ The two trials were carried out independently. A total of 10 and 8 seedlings per treatment were inoculated in trial 1 and 2, respectively. The D pathotype included isolates 19V76 and L42; the ND isolates were 19V77 and L41. ^2^ Diseased plants were determined based on VW symptoms such as wilting, leaf chlorosis and necrosis. Numbers in the parentheses indicate the percentage of disease incidence. ^3^ D plants were determined based on defoliation observed during the four weeks of the experiment. ^4^ D- and ND-positive plants were determined based on the recovery of both D and ND isolates from an inoculated plant. ^5^ All plants including the control were subjected to *V. dahliae* re-isolation individually. Numbers in parentheses indicate the frequency recovery percentage of *V. dahliae* from diseased plants. ^6^ The recovered *V. dahliae* isolates were subjected to pathotyping by duplex PCR [19].

**Table 3:** A brief description including pathotypes, single-spored status, location and year of recovery of *V. dahliae* isolates from cotton used in pathogenicity assays.

Isolates	Pathotypes ^1^	Single Spored	Location ^2^	Year of Recovery
L41	ND	Yes	Merah North, Namoi, NSW	2018
L42	D	Yes	Merah North, Namoi, NSW	2018
19V76	D	Yes	Baan baa, Namoi, NSW	2019
19V77	ND	Yes	Baan baa, Namoi, NSW	2019
L41 + L42	Mixed	Mixed	Merah North, Namoi, NSW	2018
19V76 + 19V77	Mixed	Mixed	Baan baa, Namoi, NSW	2019

^1^ Pathotypes including D and ND were designated using the duplex PCR developed by Mercado-Blanco et al. [19] ^2^ There are five main cotton growing valleys in NSW, including Gwydir, Namoi, Macquarie, Lachlan and Murrumbidgee valleys.

## Supplementary Materials

The following are available online at https://www.mdpi.com/2223-7747/9/6/750/s1, Table S1: Relative number of D and ND pathotypes^1^ recovered from the confirmed co-infected cotton plants in pot trial 1 and 2.
