# Supplementary material for: Co-Occurrence of Defoliating and Non-Defoliating Pathotypes of Verticillium Dahliae in Field-Grown Cotton Plants in New South Wales, Australia
Source: Plants (Basel). 2020 Jun 15;9(6):750. doi: 10.3390/plants9060750 (PMC7355434; doi:10.3390/plants9060750)
Supplement: Supplementary file 1 [file plants-09-00750-s001.pdf]

Supplementary data

Table S1. Relative number of D and ND pathotypes[1] recovered from the confirmed co-infected cotton plants in pot trial 1 and 2.

Co-infected Plant	Inoculated with	No. of D Isolates	No. of ND Isolates
Trial 1 – plant 1	19V76 + 19V77	1	5
Trial 1 – plant 2	19V76 + 19V77	1	5
Trial 1 – plant 3	L41 + L42	1	2
Trial 1 – plant 4	L41 + L42	6	1
Trial 2 – plant 1	L41 + L42	1	6

Seven small sections (woody chips) were excised from the collar regions of the cotton plants and plated out on sPDA for the pathogen recovery. Individual putative cultures of *V. dahliae* recovered from these sections were subjected to pathotyping using duplex PCR [19].
